# The added value of musculoskeletal ultrasound to clinical evaluation in the treatment decision of rheumatoid arthritis outpatients: physician experience matters

**DOI:** 10.1186/s12891-017-1747-2

**Published:** 2017-09-11

**Authors:** C. Sifuentes-Cantú, I. Contreras-Yáñez, L. Saldarriaga, A.C. Lozada, M. Gutiérrez, V. Pascual-Ramos

**Affiliations:** 1 Department of Immunology and Rheumatology. Instituto Nacional de Ciencias Médicas y Nutrición Salvador Zubirán, Vasco de Quiroga 15, colonia sección XVI, Tlalpan, 14000 México City, Mexico; 2 Division of Musculoskeletal Ultrasound. Instituto Nacional de Rehabilitación y Ortopedia, Calzada México-Xochimilco 289, Arenal de Guadalupe, 14389 México City, Mexico

**Keywords:** Ultrasound impact, Ultrasound value in Rheumatoid Arthritis treatment decisions

## Abstract

**Background:**

Musculoskeletal ultrasound improves the accuracy of detecting the level of disease activity (DA) in RA patients, although its impact on the final treatment decision in a real clinical setting is uncertain. The objectives were to define the percentage of clinical scenarios from an ongoing cohort of RA outpatients in which the German Ultrasound Score on 7 joints (GUS-7) impacted the treatment and to explore if the impact differed between a senior rheumatologist (SR) vs. a trainee (TR).

**Methods:**

Eighty-five consecutive and randomly selected RA outpatients underwent 170 assessments, 85 each by the SR and the TR. Initially, both physicians (blinded to each other) performed a rheumatic assessment and recommended a preliminary treatment. Then, the patients underwent the GUS-7 evaluation by an experienced rheumatologist blinded to clinical evaluations; selected joints of the clinically dominant hand were assessed by gray-scale and power Doppler (PD). In the final step, the TR and the SR integrated the GUS-7 findings with their previous evaluation and reviewed their recommendations. The patients received the final recommendation from the SR to avoid patient confusion. The study was approved by the Internal Review Board and all the patients signed informed consent. GUS-7 usefulness was separately evaluated by the SR and the TR according to a visual analogue scale (0 = not useful at all, 10 = very useful). Descriptive statistics were used.

**Results:**

The patients were primarily middle-aged females (91.4%) with (mean ± SD) disease duration of 7.5 ± 3.9 years. The majority of them (69.2% according to TR and 71.8% to SR) were in DAS28-ESR-remission. In 34 of 170 clinical scenarios (20%), the GUS-7 findings modified the final treatment proposal; 24 of these scenarios were determined by the TR vs. 10 by the SR: 70.5% vs. 29.5%, *p* = 0.01. Treatment changes (increase, decrease and joint injection) were similar between both specialists. As expected, the TR rated the GUS-7 usefulness higher than the SR, particularly in the clinical scenarios where the GUS-7 findings impacted treatment.

**Conclusions:**

Musculoskeletal ultrasound added to standard rheumatic assessments impacted the treatment proposal in a limited number of patients; the impact was greater in the TR.

## Background

Disease activity is a central aspect in the evaluation of patients with rheumatoid arthritis (RA) because it comprises signs and symptoms of the disease, impacts patient reported outcomes and is responsible for the progression of joint damage [1, 2]. Disease activity is reversible, and its abolishment or reduction to desirable levels is the major target of any therapeutic intervention. In clinical practice, it is advisable to regularly evaluate disease activity; a core set of clinical measures [3–5] and pooled indices that combine a variable number of disease activity measures [6] are available and have shown to be beneficial in following RA patients.

In 2013, the EULAR task force on imaging in RA clinical practice [7] developed 10 recommendations on various aspects of imaging in RA using research-based evidence, expert opinions and recommendations aimed to address clinical questions relevant to current practice and stated that ultrasound was a valuable candidate to detect clinical joint inflammation (superior to clinical examination), monitor disease activity, predict progression and therapeutic response and detect damage and persistent inflammation even when clinical remission was achieved. These recommendations summarized the experience gained in routine clinical practice over more than two decades, in which ultrasound was efficiently incorporated as a bedside method to enable clinical evaluation and therapy monitoring with high patient acceptability; novel scores that evaluated a reduced number of joints had been tested and were found to effectively reflect overall joint inflammation in RA, in addition to being less time consuming [8–12]. In this clinical context, the German Ultrasound 7 Score (GUS-7) combines soft tissue changes as well as erosive bone lesions in a single ultrasound scoring system [12]; the score concentrates on a small number of joint regions and examination time is reduced to approximately 10–20 min, making it a suitable candidate to integrate into daily rheumatologic practice.

It is generally accepted that disease activity is the most important factor that determines treatment consideration during routine evaluation of an outpatient with RA, although additional factors, such as comorbidities [13], costs/availability of DMARDs [14] and treatment related adverse/events, are increasingly recognized [15]. In addition, although maintained disease activity or remission status might prompt the clinician to suggest a medication change, patient’s willingness to change treatment might affect the final proposal [16]. In a study designed to capture patients with moderate to high disease activity that would prompt a discussion of medications, only in 39% of the study visits did the clinician report a medication change [17].

Ultrasound assessment has certainly improved our accuracy to detect (the level of) disease activity in RA patients, although its real impact on the final treatment proposal in real world practice has not been assessed. The primary objective of this study was to define the percentage of routine clinical scenarios in which GUS-7 findings impacted the treatment indicated by the rheumatologist in an ongoing cohort of early (at cohort inclusion) RA outpatients; we were particularly interested in exploring if the impact differed among rheumatologists categorized by experience, senior (SR) vs. trainee (TR). Additional objectives were to describe GUS-7 findings in our cohort of patients, compare GUS-7 usefulness and factors that impact treatment among physicians and explore GUS-7 patient acceptance.

## Methods

### Study population

Patients invited to participate belonged to the early arthritis clinic (EAC) of the Instituto Nacional de Ciencias Médicas y Nutrición Salvador Zubirán, a referral center for Rheumatic Diseases in México City. When first evaluated in the EAC, patients had a disease duration of < 1 year and no specific rheumatic diagnosis except for RA. Patients were evaluated every two months during the first two years of follow-up and every two, four or six months (fixed for all patients from the baseline evaluation) thereafter, depending on the patient and the disease characteristics. Treatment was prescribed by the rheumatologist in charge of the clinic and was treat-to-target oriented (T2 T). Traditional DMARDs were used in 99% of the patients with/without corticosteroids (50% of the patients received low doses of oral corticosteroids during their follow-up). In November 2015, when the study was initiated, the cohort comprised 180 RA patients with variable follow-up recruited from 2004 onward. From November 2015 to May 2016, 87 randomly selected patients from the EAC were invited to participate; 2 of these patients denied because they had time constraints. Finally, 85 patients agreed to have study evaluations and were informed about the whole process of the study.

### Study evaluations

Patients included had 2 clinical assessments; one assessment was performed by the SR in charge of the EAC and the other assessment was performed by a TR, and both were blinded to each other’s evaluation. Completed clinical assessments included 66/68 swollen/tender joint counts, acute reactant-phase determination (both erythrocyte sedimentation rate [ESR] and C reactive protein [CRP]) and a patient visual analogue scale for overall disease activity; the DAS 28 was obtained [18]. After the clinical assessments, each physician was directed to write a treatment proposal on a standardized format. Clinical charts with information regarding comorbidities, previous treatment and serious adverse events were available for both the TR and the SR.

Then, each patient had GUS-7 performed by an experienced rheumatologist trained in musculoskeletal ultrasound and blinded to the clinical evaluations. The findings were immediately recorded on standardized formats and shared with both clinicians, who were instructed to review their previous treatment proposal and confirm/change the proposal on the standardized format (blinded to each other’s proposal). In addition, both physicians were instructed to rate on a 0 to 100 mm scale, the GUS-7 usefulness for the final treatment proposal (were 100 indicates the maximum usefulness) and to select (and rate) which of the following factors was/were determinant in the final treatment proposal: clinical assessments, GUS-7, comorbidities, treatment related adverse events, costs/availability, patient’s preference and DMARD maximum dose.

Finally, only the SR met with each patient and gave him/her the final treatment recommendation.

### GUS-7 assessments

Ultrasound assessments were performed using a General Electric Logiq E ultrasound machine equipped with a high-frequency (8–18 MHz) linear transducer. All joints were scanned using a multiplanar technique, adopting the European League Against Rheumatism (EULAR) guidelines [19]. Ultrasounds were performed by a senior rheumatologist experienced in musculoskeletal ultrasound (at least 10 years of experience) who was blinded to the clinical evaluations performed by the SR and the TR, with the patient seated with hands lying in prone position (for wrist, metacarpophalangeal and proximal interphalangeal joints examination) and the patients in supine position with the legs bent at the knee (for metatarsophalangeal joints examination). All joint regions were assessed by gray-scale and power Doppler (PD), as previously published.

The following joints were assessed during the GUS-7: the wrist, second and third metacarpophalangeal (MCP2 and MCP3), second and third proximal interphalangeal joints (PIP2 and PIP3), and the second and fifth metatarsophalangeal joints (MTP2 and MTP5) of the clinically dominant side. During the GUS-7, the wrist was examined for synovitis and tenosynovitis from the dorsal, palmar and ulnar aspects; the MCP2 and MCP3 joints were evaluated for synovitis and tenosynovitis from the palmar view. Erosions were detected from the dorsal, palmar and radial (for MCP2 joint) aspects or from the dorsal and palmar aspects (for MCP3 joint). The PIP2 and PIP3 joints were assessed for synovitis from the palmar aspect and for erosions from the dorsal and palmar aspects [12]. The MTP2 and MTP5 joints were examined for synovitis from the dorsal aspect, and erosions were detected from the dorsal and palmar aspects for MTP2 joint and from the dorsal, plantar and lateral aspects for MTP5 joint. Synovitis by GUS-7 was described semiquantitatively as absence (=0), mild (=1), moderate (=2) and severe (=3) (see definitions below). Tenosynovitis and erosions were registered as being absent or present (see definitions below).

PD was performed for synovitis and tenosynovitis from the palmar and dorsal aspects in each region evaluated except for the MTP joints, which were evaluated from the plantar aspect. PD activity for synovitis and tenosynovitis were semi-quantitatively scored from grade 0 to grade 3 (see definitions below).

All the documentation was obtained on standardized formats. The GUS-7 examination of each patient took 10 to 20 min. Immediately after the GUS-7 was performed, the patients were instructed to complete a questionnaire that evaluated the following items according to a Likert scale: pain (new or increase) related to GUS-7, convenience of GUS-7 duration, patient satisfaction with GUS-7, patient preference for disease activity assessments (GUS-7 vs. clinical assessment) and patient disposition to have GUS-7 included in their routine evaluations.

### Definitions

*Clinical disease activity* was graded by the following classification criteria: DAS28 ≤ 2.6 as clinical remission, DAS28 ≤ 3.2 as mild disease activity, DAS28 ≤ 5.2 as moderate disease activity and DAS28 > 5.2 as high disease activity [20].

*Erosion*: bone surface interruption in 2 perpendicular planes [21].

*Tenosynovitis*: hypoechoic/anechoic thickened tissue with or without fluid within the tendon sheath [21].

*Grade 1 synovitis:* small hypoechoic/anechoic line beneath the joint capsule; *grade 2 synovitis:* joint capsule elevated, parallel to the joint area; *synovitis grade 3:* strong distention of the joint capsule [21].

*PD ultrasound activity:* grade 0 = no intra-articular color signal, grade 1 = up to 3 color signals or 2 single and 1 confluent signal in the intraarticular area, grade 2 = greater than grade 1 to < 50% of the intraarticular area filled with color signals, and grade 3 = ≥50% of the intraarticular area filled with color signals [21, 22].

*GUS-7 disease activity* was defined as present if ≥grade 1 PD activity was detected in at least one joint/area examined.

### Statistics

A sample size of 84 pairs of evaluations was calculated, assuming a difference between both physicians of at least 40% in the proportion of evaluations where GUS-7 impacted the treatment, with a 95% two-side confidence level and 80% power [23–26].

We performed a descriptive statistical analysis, presenting frequencies for categorical variables and measures of position and dispersion for numerical variables. The Mann-Whitney test was used to compare continuous variables, and Chi-squared and Fisher’s exact test were used to compare proportions. The weighted kappa coefficient was used to establish the agreement between the SR and the TR for level of disease activity.

The statistical analysis was performed using SPSS IBM V.21.

## Results

### Characteristics of the patients evaluated

The patients included had 170 clinical assessments performed, 85 each by the TR and the SR. At study inclusion, the patients were primarily middle-aged females with substantial follow-up, although they had short disease duration at cohort inclusion ([mean ± SD] disease duration of 5.7 ± 2.5 months); the majority of the patients had disease-specific autoantibodies as summarized in Table 1 and up to 49% had at least one comorbid condition.

**Table 1:** Population characteristics at study inclusion

N° (%) of female	77 (90.6)
Age, (mean ± SD), years	44.9 ± 12.2
Formal education, (mean ± SD), years	11.9 ± 4.2
N° (%) of patients RF+	78 (91.8)
N° (%) of patients ACCP+	76 (89.4)
Follow-up at the EAC, (mean ± SD), years	7.5 ± 4.1
N° (%) of patients with disease duration <5 years	31 (36.5)
N° (%) of patients with disease duration within 5 to 10 years	28 (32.9)
N° (%) of patients with disease duration >10 years	26 (30.6)

*N°* number, *SD* standard deviation, *RF* rheumatoid factor, *APCC* antibodies to cyclic citrullinated peptides, *EAC* early arthritis clinic

### Patients level of disease activity at study entry (Table 2)

The majority of the patients were classified as in remission according to the DAS28-ESR, although all 4 levels of disease activity (remission, low, moderate and high disease activity) were represented. There was a good correlation between the SR and the TR in patient disease activity level, with kappa = 0.822, *p* ≤ 0.0001.

**Table 2:** DAS28-ESR disease activity level according to the SR and the TR

	SR assessments (*N* = 85)	TR assessments (N = 85)	Agreement (%)
N° (%) of patients with remission	59 (69.4)	61 (71.8)	98.3
N° (%) of patients with low disease activity	8 (9.4)	7 (8.2)	71.4
N° (%) of patients with moderate disease activity	15 (17.6)	15 (17.6)	86.7
N° (%) of patients with high disease activity	2 (2.4)	2 (2.4)	66.7

*DAS28-ESR* disease activity score (28 joints evaluated)-erythrocyte sedimentation rate, *N* number, *SR* senior rheumatologist, *TR* trainee in rheumatology

### GUS-7 findings

**Fig. 1:**
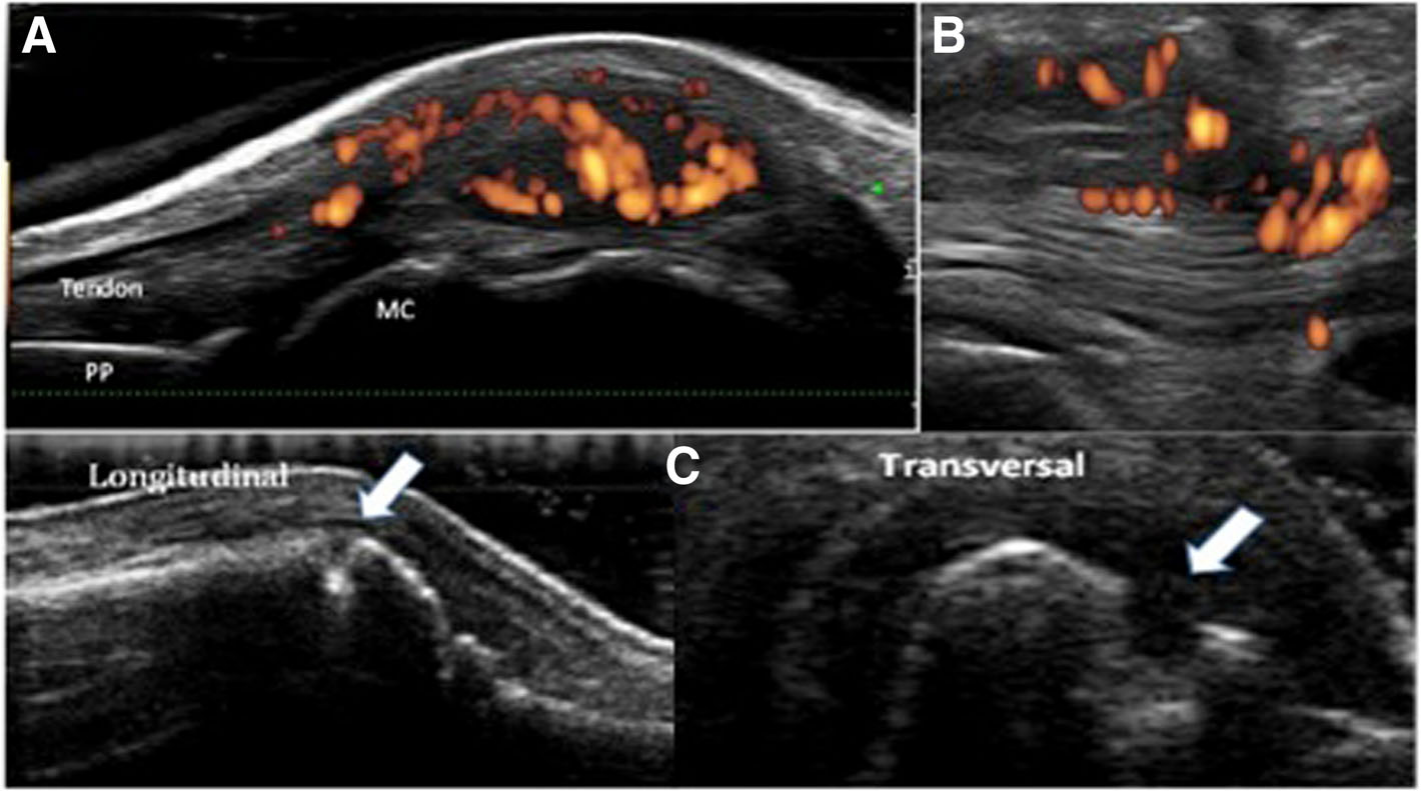
**a**. Synovitis at the MCP joint of the clinically dominant hand. Dorsal view in a longitudinal scan of the MCP joint. A moderate joint cavity widening with signs of synovial proliferation and PD signal (grade 3) is appreciated. MC = metacarpal head; PP = proximal phalanx; Tendon = common digital extensor tendon. **b**. Tenosynovitis at the first extensor compartment of the clinically dominant hand. Longitudinal scan that shows the image with PD technique, where an inflammatory process of the intra-synovial sheath consistent with tenosynovitis is appreciated along with a correlation with the area in the tendon and the anatomical damage. **c**. Bone erosion at the MC2 head. Longitudinal (*upper image*) and transversal (*bottom image*) planes. There is a bone surface interruption (*arrow*) in 2 perpendicular planes (longitudinal and transversal)

Table 3 summarizes the relevant findings. All the patients but one had at least some degree of synovitis on gray-scale US in at least one joint; the MTP2 joint was the most frequently involved, in 84.7% of the patients; the (mean ± SD) number of joints/patient with gray-scale synovitis was 3.2 ± 1.5, almost half of the patients had grade 2 synovitis, and 19 (23%) showed PD activity (Fig. 1-a); the most frequently affected joint was the MTP2 in 94.7% of the patients showing PD activity. In addition, one-third of the patients had tenosynovitis, although few (12%) had PD activity (Fig. 1-b). Finally, 33 patients (38.8%) had erosions detected by the GUS-7, and the most frequently affected bones were the MC2 and MT5 heads, each in 18.1% of the patients (Fig. 1-c).

**Table 3:** Description of GUS-7 findings

Synovitis	
N° (%) of patients with synovitis in ≥ 1 joint (grey scale)	84 (98.8)
(Mean ± SD) N° of joints/patient with synovitis (grey scale)^a^	3.2 ± 1.5
N° (%) of patients with grade 1 synovitis^a^	24 (28.6)
N° (%) of patients with grade 2 synovitis^a^	43 (51.2)
N° (%) of patients with grade 3 synovitis^a^	19 (22.6)
N° (%) of patients with synovitis (greys scale) and PD activity^a^	19 (22.6)
(Mean ± SD) N° of joints/patient with synovitis and PD activity^a^	1.6 ± 1
Tenosynovitis	
N° (%) of patients with tenosynovitis	25 (29.4)
(Mean ± SD) N° of tendons/patient with tenosynovitis (grey scale)^b^	1.5 ± 0.7
N° (%) of patients with tenosynovitis and PD activity^b^	3 (12.2)
(Mean ± SD) N° of tendons/patient with tenosynovitis and PD activity^b^	2 ± 1
Erosions	
N° (%) of patients with erosions (in ≥ 1 joint)	33 (33.8)
(Mean ± SD) N° of joints/patient with erosions^c^	1.6 ± 1

*GUS-7* German ultrasound score on 7 joints, *N°* Number, *SD* Standard deviation, *PD* Power Doppler

^a^Among 84 patients with synovitis in at least 1 joint

^b^Among 25 patients with synovitis in at least 1 joint

^c^Among 33 patients with at least one erosion in at least 1 joint

Twenty patients were classified with GUS-7 active disease, 19 of them based on PD activity on at least one joint and the other patient based on the presence of tenosynovitis with PD activity.

### GUS-7 impact on treatment

In 34 (20%) clinical scenarios (among 170 assessments), the GUS-7 findings impacted treatment; treatment changes (after GUS-7 findings were incorporated to clinical findings) consisted of an increase in 24 (70.6%) scenarios, a decrease in 8 (23.5%) and joint injection with corticosteroids in 2 (5.9%).

A total of 24 of the 34 clinical scenarios with GUS-7 treatment impact were determined by the TR vs. 10 determined by the SR: 70.5% vs. 29.5%, *p* = 0.01. In 18 clinical scenarios (52.8%) the TR and the SR agree in their decision to modify the treatment after GUS-7. Treatment changes (increase, decrease and joint injection) were similar among both physicians (data not shown). There was a good correlation between the SR and the TR in the treatment indicated (kappa = 0.645, *p* ≤ 0.0001) although the incorporation of GUS-7 findings did not improve it (kappa = 0.474, *p* ≤ 0.0001).

Finally, we compared demographic characteristics (gender, age, education), disease characteristics (rheumatoid factor, antibodies to cyclic citrullinated proteins, disease duration, DAS28, ESR, CRP, disease activity status), comorbidities and treatment (corticosteroids use and DMARDs/patient) between patients in whom the GUS-7 findings modified the treatment and their counterpart; no differences were found in the variables examined (data not shown).

### Comparison of GUS-7 usefulness between the SR and the TR

Table 4 summarizes the VAS scores from the SR and the TR. As expected, the TR rated the GUS-7 usefulness higher than the SR, particularly in the clinical scenarios where the GUS-7 findings impacted treatment. This finding was replicated within the SR GUS-7 usefulness scores.

**Table 4:** Comparison of GUS-7 usefulness VAS-scores between the SR and the TR

	SR VAS-score^a^	TR VAS-score^a^	*p*
Usefulness score among all the clinical scenarios	4.1 ± 1.9	4.9 ± 2.5	0.023
Usefulness score among clinical scenarios where GUS-7 impacted treatment	7.2 ± 0.9	8.4 ± 1.3	0.011
Usefulness score among clinical scenarios where GUS-7 did not impact treatment	3.7 ± 1.5	3.6 ± 1.3	0.47

*GUS-7* German ultrasound score on 7 joints, *SR* Senior rheumatologist, *TR* Trainee in rheumatology, *VAS* Visual analogue scale

^a^Data presented as (mean ± SD)

**Fig. 2:**
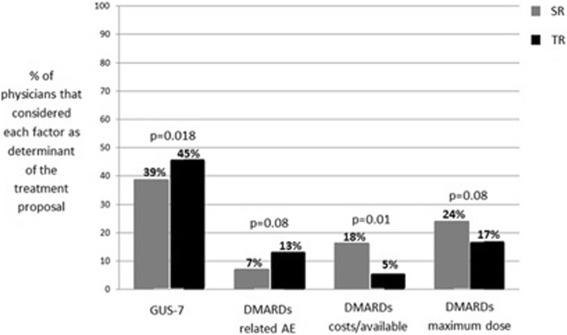
Comparison of the percentage of SR and TR that considered GUS-7, DMARD related AE, DMARD costs/availability and DMARD maximum doses as determinant in the treatment proposal

The GUS-7 was rated as a determinant in the final treatment proposal in 84.7% of the clinical scenarios after clinical assessment, which was rated as determinant in all of the clinical scenarios; the DMARD maximum dose was rated in 41.2%, comorbidities in 23.5%, DMARD cost/availability in 21.2%, DMARD-related adverse events in 20% and patient preference was rated as determinant in 14.1% of the clinical scenarios. The SR and the TR differed in the selection of the factors they considered determinant for the treatment proposal, as shown in Fig. 2; GUS-7 and DMARD-related adverse events were more frequently considered determinant in the treatment proposal by the TR, and the opposite trend was true for the SR regarding DMARD cost/availability and DMARD maximum doses.

### Patient satisfaction with GUS-7

The majority of the patients reported that the GUS-7 was painless (97.6%), the duration of the assessment was appropriate (84.7%), they were satisfied with the ultrasound assessment (95.3%), they agreed that the GUS-7 should be part of routine clinical assessments (76.5%) and they confirmed their willingness to have future ultrasound assessments (100%).

## Discussion

In the present study, we assessed the impact of adding musculoskeletal ultrasound information to the treatment decisions in an ongoing cohort of early RA outpatients and evaluated if the impact differed according to the experience of the rheumatologist involved in the treatment decision, SR vs. TR; we also compared how both physicians rated additional factors that might impact treatment decisions; finally, we explored patient satisfaction with the radiological assessment.

The study was performed in a real clinical setting of an ongoing cohort of early (at cohort inclusion) RA patients who had been treated since the beginning of their enrollment according to a T2 T strategy with traditional DMARDs (with/without corticosteroids) following current recommendations [27]; in addition, up to 49% of the patients had comorbidities. The simultaneous presence of multiple pathological conditions is more a rule than an exception in RA patients and has important academic issues and implications in daily practice [13]. We consider our results contribute to define the impact of ultrasound in RA patients’ clinical care. Additional strengths of the study were the blinding for ultrasound evaluations and for clinical assessments (between the TR and the SR).

Four years ago, the Targeted Ultrasound Initiative (TUI) group stated that targeting therapy to PD activity provided superior outcomes in RA patients compared with treating to clinical targets alone [28] and undertook an international study in 8 countries to determine the added value of musculoskeletal ultrasound to the state-of-the-art management of RA, the Targeted Ultrasound in RA (TURA) study; the results of the TURA study have not been published yet. In the present study, we found that musculoskeletal ultrasound impacted the treatment decision in a limited number of the clinical scenarios, 20%, and the impact was greater for the lesser experienced rheumatologist, the TR, compared to the SR. The role of ultrasound in RA management has been recently revisited [29], and the need to counterbalance the expanded scientific literature on the generalized benefits of ultrasound in RA management with appropriate strategy trials has been addressed. Two recent studies performed in early RA populations [30, 31] highlighted discrepancies between the potential benefits of adding ultrasound information to the treatment decisions in early RA patient management and the actual impact on clinical and imaging outcomes. In addition, although musculoskeletal ultrasound provides a more accurate additional (to clinical assessment) method for assessing disease activity, its incorporation to a T2 T strategy in early RA patients modified only 29% of all DAS-28-based DMARD decisions and did not impact patient outcomes [32]; this percentage is close to that recently reported by Diaz-Torné et al. [33], who assessed RA patients with a longer disease duration (mean follow-up of 15.5 ± 10.7 years), the majority of them treated with conventional DMARDs (68%); in their study, ultrasound information made a change in the therapeutic decision in 32% of the patients. The above mentioned studies are consistent and highlight the complexity of RA patient management that goes far beyond the assessment of disease activity and even in the clinical context of remission; a careful assessment of the risk-benefit of targeting ultrasound remission also needs to be performed. As recently highlighted by van der Heijde D [34], the ultimate target should be better long-term patient-reported outcomes. We also found that the impact was greater in the TR than in the more experienced rheumatologist. This finding could be related to a greater experience of the SR in early RA and a deeper knowledge of the patients from the clinic, so the SR can be more confident in deciding treatment based on a limited number of traditional medical and serological factors. In addition, young rheumatologists have identified training on novel imaging technologies as among the most important educational needs [35]; a more accurate understanding of novel technology might favor a rapid incorporation to routine clinical practice. Nonetheless, it should be emphasized that both clinicians (the TR and the SR) completed a 12-h course of musculoskeletal ultrasound in rheumatic diseases and that in addition to the GUS-7, 3 other factors among 6 were differently rated by the TR and the SR to impact the treatment decision.

Both physicians agreed on the impact on the treatment proposal in 3 factors (the clinical evaluation of disease activity according to DAS28, the presence and type of comorbidities and the patients’ preferences) and disagreed on the other 3 (the DMARD cost/availability, a tendency to see DMARD-related adverse events and DMARD maximum doses), although the physicians had access to the same patient medical information. The management plan for RA might be a relatively simple task if only disease activity is considered but might become more complex when additional factors are considered; in particular, patients from Latin-America share particular sociodemographic and clinical characteristics that impact their access to health care and commitment with treatment that need to be included in the treatment equation during routine clinical practice [36, 37]. In addition, considerable variation has been observed in doctors’ decisions, and these variations are known to depend on a physician’s medical characteristics and medical experience [38].

In accordance to a greater impact of the ultrasound findings in the TR’s treatment decision vs. the SR’s decision, the TR rated the GUS-7 usefulness significantly higher than the SR in the totality of the clinical scenarios and in those where the GUS-7 impacted the final recommendation. Additionally, both physicians agreed on a higher GUS-7 usefulness in those scenarios where ultrasound findings did impact the treatment. These results suggest that the SR did not discard a priori the potential benefits of incorporating musculoskeletal ultrasound findings in the treatment decision; they rather confirmed that ultrasound assessments were among the most important factors included in the final management proposal.

In the present study, the GUS-7 findings were similar to those previously described in RA patients in whom gray-scale synovitis is frequently observed, even in patients with clinical remission, as were the majority of our patients [39]; tenosynovitis was less frequently identified and confirmed previous publications [12, 40]; erosions were identified in 34% of our patients, similar to what was observed in RA patients (43%) with disease duration proxy to that of our patients [12]; erosions were frequently located in the clinically dominant MTP5 (in addition to the MCP2) as previously published [41]. Interestingly, we found that the MTP2 was the joint most frequently scored with gray-scale synovitis and PD activity; we are unaware of similar findings, although erosions have been frequently described as located in the MTP2 head [41], and there is an association between PD ultrasound activity [42], anatomical and biomechanical factors [43], and a higher risk of erosive disease; our population had a (mean ± SD) body mass index of 25.9 ± 1.8, and 22% were obese.

Finally, patients were highly satisfied with the ultrasound assessment, with minimum discomfort, adequate time requirements and agreed to have ultrasound incorporated to routine clinical assessments, although they did not pay for the study; previous studies have also shown that musculoskeletal ultrasound favors patients’ RA knowledge and their adherence to medication [44, 45].

Limitations of the study need to be addressed. First, a 40% difference between the TR and the SR in the percentage of treatment modification after GUS-7 was estimated; the difference between both physicians was 17%, and the 85 pairs of assessments performed could have been underpowered to test the primary objective. With the sample studied, the study had a 78.2 power to detect the primary objective. Second, the majority of the clinical scenarios described corresponded to remission status, some levels of disease activity (such as high DA level) were underrepresented, and the results might not be generalized to patients with such clinical status; nonetheless, in routine clinical practice, RA outpatients are expected to be in remission or to have low disease activity. Third, an important variable of the GUS-7 evaluation to impact the treatment proposal was the identification of DA; there is no consensus on the optimal scoring system for ultrasound in rheumatoid arthritis [46]. Additionally, DA definition was based on PD activity on at least one area, and low-grade PD signal might not necessarily reflect active synovitis [39]. Fourth, the GUS-7 assessed a limited number of joint sets compared to the clinical evaluation, although the existing literature confirms that they perform as well as extended joint sets [10, 47]. Finally, our study does not assess the adequacy of the final treatment in the ultimate terms of better disease and patient-reported outcomes. We are currently performing a study aimed to address the topic (Ultrasound impact in Rheumatoid Arthritis patient-reported outcomes [ULTRAPRO], ClinicalTrials.gov Identifier: NCT03228342).

## Conclusions

Disease activity is important for the treatment decision during RA patient assessment; in routine clinical practice, additional factors also need to be considered. Musculoskeletal ultrasound added to “traditional rheumatic assessments” impacted the treatment proposal in a limited number of RA outpatients, most of them were classified with remission and low disease activity; the impact was greater in the trainee in rheumatology, who also scored better ultrasound usefulness compared to the senior rheumatologist. Both physicians differed in the impact of additional factors in the final treatment proposal.

## Significance and innovations:

- Musculoskeletal ultrasound added to “traditional rheumatic assessments” impacts the treatment decision in 20% of RA outpatients.
- The impact of musculoskeletal ultrasound in the treatment decision of RA outpatients is limited for more experienced rheumatologists.
- Additional factors to disease activity determine treatment consideration during routine evaluation of an outpatient with RA. Physician experience impacts how those factors are rated.

## Abbreviations

ACCP: Antibodies to cyclic citrullinated peptides
CRP: C reactive protein
DA: Disease activity
DAS 28: Disease activity score of 28 joints
DMARD: Disease-modifying anti-rheumatic drug
EAC: Early arthritis clinic
ESR: Erythrocyte sedimentation rate
EULAR: European League Against Rheumatism
GUS-7: German ultrasound score on 7 joints
MCP: Metacarpophalangeal joint
MTP: Metatarsophalangeal joint
PD: Power doppler
PIP: Proximal interphalangeal joint
RA: Rheumatoid arthritis
RF: Rheumatoid factor
SD: Standard deviation
SR: Senior rheumatologist
T2 T: Treat-to-Target
TR: Trainee in rheumatology
TUI: Targeted ultrasound initiative
TURA: Targeted ultrasound in RA study
VAS: Visual analogue scale
